# Polymerase delta-interacting protein 2 deficiency protects against blood-brain barrier permeability in the ischemic brain

**DOI:** 10.1186/s12974-017-1032-1

**Published:** 2018-02-17

**Authors:** Marina S. Hernandes, Bernard Lassègue, Lula L. Hilenski, Jonathan Adams, Ning Gao, Chia-Yi Kuan, Yu-Yo Sun, Lihong Cheng, Daniel S. Kikuchi, Manuel Yepes, Kathy K. Griendling

**Affiliations:** 1 Division of Cardiology, Department of Medicine, Emory University, 101 Woodruff Circle, 308 WMB, Atlanta, GA 30322 USA; 2 Division of Endocrinology, Metabolism and Lipids, Department of Medicine, Emory University, Atlanta, GA 30322 USA; 3 Division of Neurology, Department of Pediatrics, Emory University, Atlanta, GA 30322 USA; 4 Department of Neurology, Emory University, Atlanta, GA 30322 USA; 5 Division of Neuroscience, Yerkes National Primate Research Center, Emory University, Atlanta, GA 30329 USA

**Keywords:** Poldip2, Cerebral ischemia, Blood-brain barrier, Cytokines, Astrocytes

## Abstract

**Background:**

Polymerase δ-interacting protein 2 (Poldip2) is a multifunctional protein that regulates vascular extracellular matrix composition and matrix metalloproteinase (MMP) activity. The blood-brain barrier (BBB) is a dynamic system assembled by endothelial cells, basal lamina, and perivascular astrocytes, raising the possibility that Poldip2 may be involved in maintaining its structure. We investigated the role of Poldip2 in the late BBB permeability induced by cerebral ischemia.

**Methods:**

Transient middle cerebral artery occlusion (tMCAO) was induced in Poldip2^+/+^ and Poldip2^+/−^ mice. The volume of the ischemic lesion was measured in triphenyltetrazolium chloride-stained sections. BBB breakdown was evaluated by Evans blue dye extravasation. Poldip2 protein expression was evaluated by western blotting. RT-PCR, zymography, and ELISAs were used to measure mRNA levels, activity, and protein levels of cytokines and MMPs. Cultured astrocytes were transfected with Poldip2 siRNA, and mRNA levels of cytokines were evaluated as well as IκBα protein degradation.

**Results:**

Cerebral ischemia induced the expression of Poldip2. Compared to Poldip2^+/+^ mice, Poldip2^+/−^ animals exhibited decreased Evans blue dye extravasation and improved survival 24 h following stroke. Poldip2 expression was upregulated in astrocytes exposed to oxygen and glucose deprivation (OGD) and siRNA-mediated downregulation of Poldip2 abrogated OGD-induced IL-6 and TNF-α expression. In addition, siRNA against Poldip2 inhibited TNF-α-induced IκBα degradation. TNF-α, IL-6, MCP-1, VEGF, and MMP expression induced by cerebral ischemia was abrogated in Poldip2^+/−^ mice. The protective effect of Poldip2 depletion on the increased permeability of the BBB was partially reversed by systemic administration of TNF-α.

**Conclusions:**

Poldip2 is upregulated following ischemic stroke and mediates the breakdown of the BBB by increasing cerebral cytokine production and MMP activation. Therefore, Poldip2 appears to be a promising novel target for the development of therapeutic strategies to prevent the development of cerebral edema in the ischemic brain.

**Electronic supplementary material:**

The online version of this article (10.1186/s12974-017-1032-1) contains supplementary material, which is available to authorized users.

## Background

Polymerase δ-interacting protein 2 (Poldip2, also known as PDIP38 and mitogenin 1) is a multifunctional protein originally identified as a human DNA polymerase interacting protein [1]. Several recent studies suggest that Poldip2 mediates many cellular functions such as mitochondrial morphology [2, 3], DNA replication and repair [4], reactive oxygen species generation via activation of NADPH oxidase-4 [5], cytoskeletal remodeling, and cell migration [5], as well as cell cycle progression [6]. We have recently demonstrated that Poldip2 is necessary for integrity and function of conduit arteries [7]. Heterozygous deletion of Poldip2 reduces H_2_O_2_ production in vivo, leading to increases in extracellular matrix deposition and greater vascular stiffness and resulting in protection against aneurysms. In contrast, loss of Poldip2 decreases angiogenesis and formation of capillaries and arteries and impairs matrix metalloproteinase (MMP) activity in a hind limb ischemia model [8]. Thus, Poldip2 seems to play an essential role in vascular physiology and perhaps also in modulating vascular pathology, depending on the insult. However, its function in the brain and cerebral vasculature is completely unknown, and the cell types in which it is expressed have not been identified.

The blood-brain barrier (BBB) forms the interface between the vasculature and the brain. It is composed of endothelial cells, the basal lamina, and astrocytic end-feet processes and regulates the passage of substances from the intravascular space into the brain [9]. The permeability of the BBB is determined not only by the integrity of interendothelial tight junctions but also by the interaction between astrocytic end-feet processes, endothelial cells, and the basement membrane. Accordingly, approximately 95% of the BBB is embraced by astrocytic end-feet processes [10, 11]. The BBB is particularly sensitive to hypoxia. Specifically, cerebral ischemia is well known to increase the permeability of the BBB by perturbing interactions between components of the BBB. Early after the onset of the ischemic injury, astrocytes release pro-inflammatory cytokines that lead to degradation of the extracellular matrix by MMPs, redistribution of interendothelial tight junctional proteins, and increased permeability of the BBB with the development of cerebral edema [12, 13]. Increased production of reactive oxygen species also contributes to disruption of the BBB [14].

Because several functions of Poldip2, including regulation of reactive oxygen species, matrix remodeling, and cytoskeletal dynamics, are known to be involved in the response of the BBB to ischemic insult, we postulated that Poldip2 may represent a potential novel mechanism regulating BBB permeability. To test this hypothesis, wild-type and Poldip2 heterozygous null mice were subjected to transient middle cerebral artery occlusion (tMCAO) to induce BBB disruption. Our data show that Poldip2^+/−^ mice had reduced mortality and BBB permeability after cerebral ischemia. Our results further suggest that increased expression of Poldip2 following the onset of cerebral ischemia leads to a Poldip2-regulated pro-inflammatory response in astrocytes and likely other cell types, increasing the late-phase permeability of the BBB. Thus, Poldip2 represents a potential target to attenuate the development of edema in the ischemic brain and other neurological diseases associated with increased permeability of the BBB.

## Methods

### Animals

Poldip2 gene trap mice on the C57BL/6 background were produced by the Texas A&M Institute for Genomic Medicine (College Station, TX). A gene trap construct was inserted into the first intron of Poldip2 in mouse embryonic stem cells. The location of the gene trap was verified by polymerase chain reaction and sequencing. Mice were genotyped using a standard three-primer PCR method. Because homozygous deletion of Poldip2 is perinatal lethal, Poldip2^+/−^ mice were used for this study. Characterization of these mice has been published previously [6].

### Animal model of cerebral ischemia

Transient middle cerebral artery occlusion (tMCAO) was induced in 9- to 13-week-old male mice. Animals were anesthetized with isoflurane (oxygen delivered at 0.5 L/min with 3% isoflurane for induction and 1.5% for maintenance), and the middle cerebral artery (MCA) was occluded for 30 min with a 6-0 nylon filament inserted in the left carotid artery as described elsewhere [15]. Cerebral perfusion (CP) was monitored throughout the surgical procedure with laser Doppler (Perimed Inc., North Royalton, OH), and only animals with a > 70% decrease in CP after occlusion and complete recovery after filament removal were included in this study. Heart rate, systolic, diastolic, and mean arterial blood pressure were controlled with an IITC 229 System (IITC-Lice Science; Woodland Hills, CA).

For additional experiments, non-reperfusion cerebral ischemia was induced by temporary ligation of the right common carotid artery followed by 7.5% hypoxia induction for 30 min. The core body temperature was maintained at 37.5 ± 0.5 °C using a rectal thermoprobe coupled to a heating lamp, according to the protocol described previously [16].

### Lesion volume

Twenty-four hours after tMCAO, mice were anesthetized and perfused with ice-cold phosphate-buffered saline. The brains were removed and sliced into 2-mm coronal sections, and the volume of the ischemic lesion was measured in 2,3,5-triphenyltetrazolium chloride (TTC)-stained sections as described elsewhere [17]. The lesion volume was calculated using ImageJ.

### Behavioral testing

Motor performance was tested using an accelerated RotaRod (0207-003 M; Columbus Instruments, USA) challenge before and after sham surgery or temporary ligation of the right common carotid artery, followed by 7.5% hypoxia induction. Animals were placed on the RotaRod at a starting speed of 4 rpm, followed by acceleration to 20 rpm. Latency to fall was recorded. All animals were trained on the RotaRod in three consecutive daily sessions consisting of three trials separated by no more than 5 min. The results of the three trials were used to calculate a daily session average. The baseline measurement for each animal was calculated by averaging the results of the three RotaRod sessions preceding surgery. Identical RotaRod sessions were repeated 6 and 24 h after surgery. Motor data are presented as mean duration (three trials) on the RotaRod, expressed as percent of baseline for each animal. All behavior testing was performed in a blinded fashion.

### Real-time investigation of cerebral blood flow dynamic by laser Doppler perfusion imaging

Laser Doppler perfusion imaging (LDPI) was used to evaluate the cerebral blood flow (CBF) dynamics in Poldip2^+/+^ and Poldip2^+/−^ mice under normocapnic and hypercapnic conditions. Anesthetized animals were endotracheally intubated and attached to a mechanical ventilator (PhysioSuite, Kent Scientific) delivering gas with 2% of isoflurane. End-tidal CO_2_ pressure (PhysioSuite, Kent Scientific) was monitored, and the intact skull was scanned in the prone position using LDPI (MoorFLPI-2; Moor Instruments Inc.). Hypercapnia was induced by 5% CO_2_ inhalation (in 21% O_2_/74% N_2_) for 5 min alternating with normocapnia (21% O_2_/79% N_2_) for 5 min. Hypercapnia and normocapnia exposures were repeated three times during each experiment, and the highest CBF increase during hypercapnia was considered to be the maximum response for each animal. The baseline CBF was recorded with the MoorFLPI software in arbitrary perfusion units (flux), and the change of CBF (∆CBF%) was normalized to the baseline CBF. The cerebrovascular reactivity (CVR) was calculated as the increase of CBF (%) divided by the maximum increase in end-tidal CO_2_ pressure (∆ mmHg) during hypercapnia [18].

### Micro-CT imaging of brain vasculature

A quantitative micro-CT-based method was used for the evaluation of cerebral vasculature as previously reported [19]. Briefly, animals were anesthetized and perfused at physiological pressure through the left ventricle with papaverine hydrochloride (4 g/L) in normal saline, followed first with normal saline and second with 10% neutral buffered formalin. Animals were subsequently perfused with a lead chromate-based contrast agent (MICROFIL MV-122 Yellow, Flow Tech Inc.), which was allowed to polymerize overnight at 4 °C before the brain tissue was retrieved. The intact whole brain was removed from the cranial cavity and stored in 10% buffered formalin until micro-CT examination was performed. Brain vasculature was imaged using a micro-CT system (μCT 40, Scanco Medical; Bassersdorf, Switzerland), at 16-μm resolution (isotropic voxel size, creating 1024 × 1024 pixel slices) with a voltage of 55 kV and a current of 145 μA. Serial tomograms were reconstructed from raw data using a cone beam filtered backprojection algorithm adapted from Feldkamp et al. [20]. The noise was removed using a low-pass Gaussian filter (*σ* = 1.2, support = 2). The brains were evaluated individually to quantify the 3D histomorphometric values vascular volume, connectivity, number, and spacing between vessels. These parameters are standard for the analysis of the trabecular bone microstructure [21] and have been used previously for microvascular networks [22]. Horos medical imaging software was used to render 3D models, presented here as 2D maximal intensity projections.

### Transmission electron microscopy

Poldip2^+/−^ mice and Poldip2^+/+^ mice were anesthetized with isoflurane and perfused transcardially with 3% depolymerized paraformaldehyde and 0.15% glutaraldehyde in 0.1 M phosphate buffer. The brains were sliced into 100-μm sections using a vibratome. Sections were washed with 0.1 M phosphate buffer (pH 7.4) and post-fixed in 1% osmium tetroxide in the same buffer. Sections were dehydrated through a graded ethanol series to 100% and flat-embedded in Eponate 12 resin (Ted Pella Inc., Redding, CA). Following resin polymerization in a 60 °C oven, the cortical area of interest was isolated, re-embedded in the resin, and then placed in the oven for further polymerization. Ultrathin sections were obtained on a Leica UltraCut ultramicrotome (Leica Microsystems) at 70 nm and counter-stained with 5% aqueous uranyl acetate and 2% lead citrate. Sections were examined on a Hitachi HT-7700 transmission electron microscope (Hitachi High Technologies of America, Inc) equipped with an Advanced Microscopy Techniques CCD camera (Woburn, MA).

### BBB permeability assays

Alterations in the brain vascular permeability were determined by intraperitoneal injections of Evans blue dye (Sigma-Aldrich, St Louis, MO). Evans blue binds to serum proteins such as albumin and can be used to quantify alterations in vascular permeability, since albumin does not cross the endothelial barrier under basal physiological conditions [23, 24]. Briefly, mice were injected with 2% Evans blue solution in normal saline (4 mL/kg of body weight) 24 h following tMCAO. Evans blue was allowed to circulate for 3 h. Mice were euthanized and perfused with 50 mL of ice-cold phosphate-buffered saline. The brains were then removed and divided into ipsilateral and contralateral hemispheres. Evans blue was extracted at 55 °C overnight with formamide. Dye concentration was quantified spectrophotometrically in the supernatant at 620 nm and normalized to hemisphere weight.

### TNF-α treatment

An intraperitoneal injection of recombinant mouse TNF-α (5 μg/mouse—PeproTech Inc. Rocky Hill, USA) was administered to Poldip2^+/+^ and Poldip2^+/−^ mice immediately after tMCAO. This dose has previously been shown to increase vascular permeability [25]. Twenty-four hours after TNF-α administration, BBB permeability assays were performed.

### Western blotting

Twenty-four hours after tMCAO, animals were deeply anesthetized and perfused with 50 mL of ice-cold phosphate-buffered saline. The brains were removed, and the regions of interest quickly collected, frozen in liquid nitrogen, and stored at −70 °C until use. The tissue was homogenized at 4 °C in extraction buffer (Tris, pH 7.4, 100 mmol/L; EDTA 10 mmol/L; PMSF 2 mmol/L; aprotinin 0.01 mg/mL). Whole cell lysate was prepared from cultured astrocytes using the same extraction buffer used for the brain samples. The homogenates were centrifuged at 14,000×*g* at 4 °C for 20 min, and protein concentration of the supernatant was determined using the Bradford assay. The material was stored in sample buffer (Tris/HCl 125 mmol/L, pH 6.8; 2.5% (*w*/*v*) SDS; 2.5% 2-mercaptoethanol, 4 mmol/L EDTA, and 0.05% bromophenol blue) at − 70 °C until gel loading. Following separation by SDS-PAGE, proteins were transferred to a nitrocellulose membrane and assessed by western blotting with primary antibodies against Poldip2 (ab181841; Abcam or custom made by GenScript Corporation, Piscataway, NJ), IκBα (ab32518; Abcam), β-actin (A5441; Sigma), and β-tubulin (21465; Cell Signaling). Blots were incubated with horseradish peroxidase (HRP)-conjugated secondary antibodies depending on the species of the primary antibody [anti-mouse (NA931; GE), anti-goat (205-295-108; Jackson), and anti-rabbit (70745; Cell Signaling)] and assessed using enhanced chemiluminescence (ECL, GE). HRP-induced luminescence was detected with Amersham Hyperfilm ECL (GE). Detected bands were scanned, and densitometry was performed using ImageJ.

### RNA extraction and RT-qPCR

Total RNA was purified with the RNeasy Plus kit (Qiagen Chatsworth, CA). Reverse transcription was performed using Superscript II reverse transcriptase (Invitrogen Carlsbad, CA) with random primers, and cDNA was purified with the QIAquick kit (Qiagen). cDNA was amplified with primers against β-2 microglobulin (B2M) (5′-GGCCTGTATGCTATCCAGAA-3′, 5′-GAAAGACCAGTCCTTGCTGA-3′, annealing temperature (Ta) 55 °C), glyceraldehyde 3-phosphate dehydrogenase (GAPDH) (5′-CTGGAGAAACCTGCCAAGTA-3′, 5′-TGTTGCTGTAGCCGTATTCA-3′, 58 °C), ribosomal protein L13A (RPL) (5′-ATGACAAGAAAAAGCGGATG-3′, 5′-CTTTTCTGCCTGTTTCCGTA-3′,  58 °C), IL-6 (5′-GTCTATACCACTTCACAAGTC-3′, 5′-TGCATCATCGTTGTTCATAC-3′, 50 °C), TNF-α (5′-CTATGTCTCAGCCTCTTCTC-3′, 5′-CATTTGGGAACTTCTCATCC-3′, 52 °C), TGF-β (5′-CATCTCGATTTTTACCCTGG-3′, 5′-AAAGGTAGGTGATAGTCCTG-3′, 51 °C), MMP-2 (5′-ACAGGACATTGTCTTTGATG-3′, 5′-TACACAGCGTCAATCTTTTC-3′, 51 °C), MMP-9 (5′-CTTCCAGTACCAAGACAAAG-3′, 5′-ACCTTGTTCACCTCATTTTG-3′, 51 °C), TIMP1 (5′-CTAGAGACACACCAGAGATAC-3′, 5′-CCCATGAATTTAGCCCTTATG-3′, 51 °C, TIMP2 (′GGATTCAGTATGAGATCAAGC-3′, 5′-GCCTTTCCTGCAATTAGATAC-3′, 51 °C), IκB (5′-CAGAATTCACAGAGGATGAG-3′, 5′-CATTCTTTTTGCCACTTTCC-3′, 51 °C), MCP-1 (5′-AGCACCAGCCAACTCTCACT-3′, 5′-TCTGGACCCATTCCTTCTTG-3′, 63 °C), and VEGF (5′-CAGACCAAAGAAAGACAGAAC-3′, 5′-TACGTTCGTTTAACTCAAGC-3′, 55 °C) using Platinum Taq DNA polymerase (Invitrogen) in the presence of SYBR green I (Invitrogen). Reactions were carried out in glass capillaries, using a LightCycler 1.2 (Roche Applied Science, Indianapolis, IN) real-time thermocycler. Data analysis was performed using the mak3 module of the qpcR software library [26, 27] in the R environment [28].

### Gelatin zymography

MMP-9 levels in ischemic brain homogenates were measured by gelatin zymography as described by Toth and Fridman [29]. Mice were deeply anesthetized and transcardially perfused with ice-cold phosphate-buffered saline. The brains were dissected and placed at − 80 °C until use. Each tissue was homogenized on ice with 300 μL lysis buffer (25 mM Tris-HCl, pH 7.5; 100 mM NaCl; 1% Nonidet P-40 and 0.1% protease inhibitor cocktail (SIGMAFAST Protease Inhibitor Cocktail Tablets, EDTA-Free, Sigma). The lysates were spun at 16,000×*g* for 10 min at 4 °C, and the supernatants were collected. Total protein concentration was determined using the Bradford assay method. Thirty micrograms of lysates were loaded in each lane and separated using a 10% precast polyacrylamide gel with gelatin (Bio-Rad, Hercules, CA). After electrophoresis, the gel was incubated with Zymography Renaturating Buffer (Bio-Rad) for 30 min at room temperature, followed by incubation with Zymogram Development Buffer (Bio-Rad) for 42 h at 37 °C. After incubation, the gel was stained with SimplyBlue™ Safe Stain (Invitrogen, Carlsbad CA) for 1 h and destained with water for 6 h.

### Confocal microscopy

Mice were deeply anesthetized and subjected to transcardiac perfusion with a buffered saline solution, followed by a fixative solution containing 4% paraformaldehyde (PFA) dissolved in 0.1 mol/L phosphate buffer (PB, pH 7.4). The brains were collected, post-fixed in PFA for 4 h, and transferred to a 30% sucrose solution for 48 h in PB to ensure cryoprotection. The brains were sectioned with a cryostat. Seven micrometer-thick coronal sections were incubated for 12–16 h with anti-glial fibrillary acidic protein (GFAP) (#12389, diluted 1:300, Cell Signaling, Danvers, MA) and anti-Poldip2 (diluted 1:500, custom made by GenScript) antibodies diluted in 0.3% of Triton X-100, containing 0.05% normal donkey serum (Abcam, Cambridge, MA, ab166643). Following three washes of 10 min each with PB, sections were incubated for 2 h with secondary antibodies conjugated to specific fluorophores for detection (1:50 anti-goat Cy5.5, Abcam, ab6951 for Poldip2, and 1:50 anti-rabbit Alexa Fluor 568, Thermo Fisher Scientific, Waltham, MA, A10042 for GFAP), and Isolectin IB_4_ from *Griffonia simplicifolia* conjugated to Alexa Fluor 488 (Invitrogen/Molecular Probes Eugene, OR). Samples were mounted in Vectashield mounting medium containing DAPI (Vector Laboratories, Inc. H-1200). Images were obtained using a Zeiss LSM 510 META Laser Scanning Confocal Microscope System using a Plan-Apo 40× oil objective lens and Zeiss ZEN acquisition software. Controls with no primary antibody showed no fluorescence. To convert acquired Z stacks into a 3D projection in all planes and different angles and for making videos, Bitplane Imaris 6.4.2 was used. MP4 files were made using the open source platform VCL and MPEG Streamclip software.

### Cell culture and oxygen-glucose deprivation

C8-D1A mouse type I astrocytes (ATTC) were grown in DMEM/F12 media (Invitrogen) supplemented with 10% fetal bovine serum, 2 mmol/L L-glutamine, 100 units/mL penicillin, and 100 mg/mL streptomycin. Media was changed every 2 days until cells reached confluence. Oxygen-glucose deprivation (OGD) treatment in vitro was used to most closely resemble the in vivo hypoxic conditions as previously described [30]. Cells were washed twice and incubated in glucose-free DMEM (Invitrogen) under hypoxic conditions (0.1% O_2_/5% CO_2_/95% N_2_ at 37 °C) for 4 h. At the end of the OGD, astrocytes were incubated in their regular DMEM/F12 media and reintroduced to the regular atmospheric oxygen level for an additional 3, 6, 8, or 24 h (reoxygenation). In each experiment, cultures exposed to OGD were compared with normoxic controls supplied with DMEM/F12 containing glucose and maintained in standard incubation conditions (normoxia; 21% O_2_/5% CO_2_ at 37 °C).

### siRNA

For transfection with siRNA, astrocytes (2 × 10^6^) were transfected with mouse siPoldip2 (siPoldip2; sense: 5′-GUCUACUGGUGGCGCUAUU[dT][dT]-3′, antisense: 5′-AAUAGCGCCACCAGUAGAC[dT][dT]-3′; Sigma) or the Allstars control siRNA (siNegative; sense: 5′-GGGUAUCGACGAUUACAAAUU-3′, antisense: 5′-UUUGUAAUCGUCGAUACCCUG-3′; Qiagen) using a Nucleofector (Amaxa Biosystems) set to the T20 program. After transfection, the cells were allowed to attach on the collagen-coated substrate in complete media overnight. The following day, cell media were changed to serum-reduced media (0.1% FBS) for an additional 16 h until hypoxia experiments were performed. Gene silencing was confirmed using immunoblotting. Cells were transfected with a final siRNA concentration of 25 nmol/L.

### Multiplexed ELISAs

The brains were harvested 24 h after tMCAO, divided into ipsilateral and contralateral hemispheres, and homogenized at 4 °C in lysis buffer for ELISA (Signosis, Santa Clara, CA, USA). The homogenates were centrifuged at 20,000×*g* at 4 °C for 10 min, and the protein concentration of the supernatant was determined using the Bradford assay. The production of IL-6 and TNF-α was measured, using 75-μg supernatant per well, with a Meso Scale Discovery (MSD) custom multiplex high-sensitivity ELISA plate and the MESO QuickPlex SQ 120 high performance, electrochemiluminescence immunoassay detection system per the manufacturer’s instructions (Meso-Scale Discovery, Gaithersburg, MD).

### Statistical analyses

Data are expressed as the mean ± SEM. Statistical analyses were performed using one-way ANOVA followed by Tukey’s multiple comparison post hoc tests or 2-way ANOVA, followed by Bonferroni’s multiple comparison test as indicated in the figure legends. *p* < 0.05 was considered statistically significant. Analysis of tMCAO surgery survival was calculated using online calculators from GraphPad.com (http://graphpad.com/quickcalcs/confInterval2/). Data are expressed as % survival ± 10% confidence intervals. Confidence intervals were calculated according to the modified Wald method, and a 2 × 2 contingency table was analyzed using a two-tailed Fisher’s exact text.

## Results

### Characterization of the cerebral vasculature and cerebral blood flow following Poldip2 depletion in vivo

Micro-CT and LDPI studies were performed in order to investigate whether Poldip2 depletion affects the anatomic organization of the cerebral vasculature and cerebral blood flow, respectively. For the micro-CT studies, several morphological parameters were quantitatively assessed. No difference was observed in vascular volume (4.00 ± 0.38 vs. 4.36 ± 0.47 connections per mm^3^), vessels per unit area (0.40 ± 0.03 vs. 0.45 ± 0.03 mm^2^), mean thickness (0.096 ± 0.002 vs. 0.095 ± 0.002 mm), or spacing between vessels (2.62 ± 0.20 vs. 2.30 ± 0.20 mm), and only a small increase in connectivity (0.05 ± 0.005 vs. 0.08 ± 0.009, *p* < 0.04 mm^3^) was observed (Additional file 1: Figure S1a). Segmentation analysis of images from nine to ten mice of each genotype showed no obvious abnormalities in the Circle of Willis of Poldip2^+/−^ mice. Specifically, the distribution of the MCA branch appeared to be normal, as did the connection between the anterior and posterior cerebral arteries (Additional file 1: Figure S1b). As shown in Additional file 1: Figure S1c, no significant difference at baseline of CBF flux was observed between Poldip2^+/−^ mice and Poldip2^+/+^ mice (317 ± 50 vs. 322 ± 23 flux). Cerebrovascular reactivity (CVR) induced by hypercapnia was found to be similar between Poldip2^+/−^ mice and Poldip2^+/+^ mice (Additional file 1: Figure S1d). Hypercapnia induced by 5% CO_2_ inhalation induced a similar global increase in CBF in Poldip2^+/+^ and Poldip2^+/−^ mice (Additional file 1: Figure S1e).

In addition, electron microscopy was performed to examine the ultrastructure of small capillaries in the brain cortex. As shown in Additional file 1: Figure S1f, at baseline, the brain capillaries were intact and structurally similar between Poldip2^+/−^ mice and their littermate controls, suggesting that BBB integrity is normal.

### Poldip2 protein expression colocalizes with cortical astrocytes

As demonstrated by histological analysis of the Poldip2^+/+^ mice cortical brain sections, Poldip2 was clearly, although not exclusively, expressed in glial fibrillary acidic protein (GFAP)-expressing perivascular astrocytes (Additional file 2: Figure S2 and Additional file 3: Video S1), as well as neurons, microglia, and to a lesser extent endothelial cells (data not shown). The specificity of the Poldip2 antibody used in those experiments has been reported previously [6].

### Poldip2 protein expression is increased in ischemic brain tissue following tMCAO

**Fig. 1:**
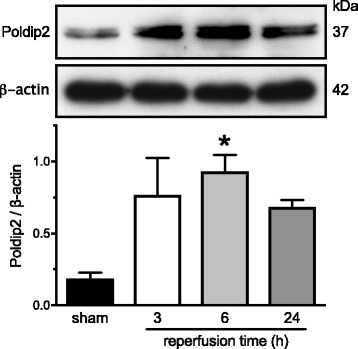
Poldip2 protein expression is upregulated in the ischemic brain tissue. Mouse brains were harvested after sham surgery or tMCAO, followed by reperfusion for the indicated time. Poldip2 was measured in the ischemic hemisphere by western blotting and densitometry. Representative blots are shown. The bar graph represents means ± SEM of four independent experiments normalized to β-actin. One-way ANOVA *p < 0.05 vs. sham

We first tested whether Poldip2 expression is regulated in ischemic brain tissue following tMCAO in wild-type mice. Poldip2 protein levels were monitored 3, 6, and 24 h after reperfusion. We found significantly increased Poldip2 protein expression in the ischemic hemisphere 6 h after reperfusion (*p* < 0.05) compared with sham-operated mice (Fig. 1).

### Poldip2 does not affect lesion volume but participates in permeabilization of the BBB during the late phase of the ischemic injury and impacts survival and motor performance

**Fig. 2:**
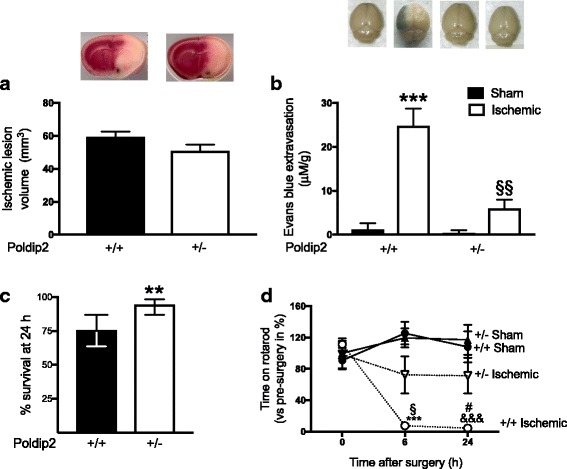
Poldip2 deletion reduces blood-brain barrier disruption, increases the survival rate and motor function 24 h after tMCAO. **a** Comparison between the mean volume of the ischemic lesion in Poldip2^+/+^ and Poldip2^+/−^ mice 24 h after tMCAO. The bar graph represents means ± SEM of 5-7 mice per group. **b** Mice received intraperitoneal injections of Evans blue 3 h before sacrifice. The brains were harvested and photographed before separation of hemispheres. Dye extravasation was quantified spectrophotometrically after overnight extraction and normalized to the corresponding hemisphere weight. The bar graph represents means ± SEM of three to six mice per group. Two-way ANOVA ***p < 0.001 vs. Poldip2^+/+^ sham and §§*p* < 0.01 vs. Poldip2^+/+^ ischemic mice. **c** Poldip2 depletion increases the survival rate 24 h after tMCAO. **p < 0.01. Bars represent % survival ± 10% confidence intervals, calculated using the modified Wald method. Postoperative deaths due to surgical complications were excluded from the data analyzed. **d** Comparison between the RotaRod performance in Poldip2^+/+^ and Poldip2^+/−^ mice 6 and 24 h after cerebral ischemia induction. Data points represent the means ± SEM (time on RotaRod vs. pre-surgery in percent) of five to six mice per group. Two-way ANOVA ***p < 0.001 vs. Poldip2^+/+^ sham 6 h; &&&*p* < 0.001 vs. Poldip2^+/+^ sham 24 h; §*p* < 0.05 vs. Poldip2^+/−^ ischemic mice 6 h and #*p* < 0.05 vs. Poldip2^+/−^ ischemic mice 24 h

To evaluate whether or not Poldip2 depletion affects the lesion volume following tMCAO, Poldip2^+/+^ and Poldip2^+/−^ brain slices were analyzed using TTC staining. Twenty-four hours after reperfusion, no significant differences were found between Poldip2^+/+^ and Poldip2^+/−^ mice (Fig. 2a).

Disruption of the architecture of the BBB during cerebral ischemia leads to neutrophil infiltration and influx of plasma proteins and, subsequently, to the formation of cerebral edema, which is responsible for much of the morbidity following stroke [31–33]. To determine if the loss of Poldip2 affects the integrity of the BBB permeability, we injected Evans blue intraperitoneally and examined its extravasation into the brain in Poldip2^+/+^ and Poldip2^+/−^ mice 24 h following reperfusion. As expected, increased Evans blue staining was observed in the brains of Poldip2^+/+^ mice, which is indicative of severe late BBB disruption following cerebral ischemia. In contrast, Poldip2^+/−^ mice were significantly protected against late BBB permeability after tMCAO. Evans blue extravasation was not observed in either Poldip2^+/+^ or Poldip2^+/−^ sham-operated mice (Fig. 2b). The decreased permeability observed in Poldip2^+/−^ mice after tMCAO was further confirmed by horseradish peroxidase extravasation (data not shown) as well as in an alternative cerebral ischemia model induced by temporary unilateral carotid artery ligation followed by 7.5% hypoxia induction (Additional file 4: Figure S3).

As less edema should correlate with better survival, we hypothesized that the loss of Poldip2 would positively influence survival. Indeed, 24 h following reperfusion, the overall survival of Poldip2^+/−^ mice was significantly higher than that of Poldip2^+/+^ mice (Fig. 2c). Next, we analyzed the impact of Poldip2 depletion on motor performance after stroke by employing the RotaRod test. Poldip2^+/−^ mice presented significantly improved motor performance 6 and 24 h following cerebral ischemia induction when compared to Poldip2^+/+^ mice (Fig. 2d).

### Astrocyte activation is suppressed following Poldip2 depletion in vivo

**Fig. 3:**
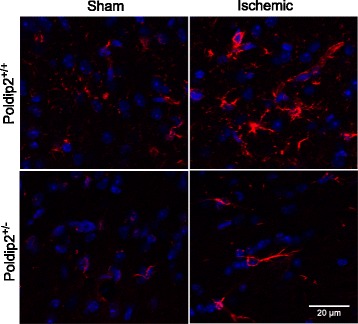
Loss of Poldip2 suppresses activation of astrocytes in the in the peri-infarct penumbra cortex. Sections were stained for immunofluorescence with primary antibodies specific for the astrocyte marker GFAP (red). Nuclei were stained with DAPI (blue). Images are representative of four independent experiments

Because astrocytes are an important component of the BBB, we examined the effect of Poldip2 depletion on astrocyte activation using GFAP, a canonical marker for astrogliosis. To test if Poldip2 regulates the astrocytic response to cerebral ischemia, Poldip2^+/+^ and Poldip2^+/−^ mice were subjected to tMCAO, and GFAP expression was evaluated in the cortex. Twenty-four hours after cerebral ischemia induction, a dramatic increase in GFAP immunoreactivity was observed in the peri-infarct penumbra cortex in Poldip2^+/+^ mice. Importantly, astrocytes appeared hypertrophic, which is indicative of their activation. Heterozygous depletion of Poldip2 abrogated the tMCAO-induced increased GFAP immunoreactivity, suggesting that Poldip2 signaling plays a role in promoting astrogliosis in the cortex (Fig. 3).

### Oxygen-glucose deprivation upregulates Poldip2 protein expression and mediates upregulation of IL-6 and TNF-α mRNA in cultured astrocytes

**Fig. 4:**
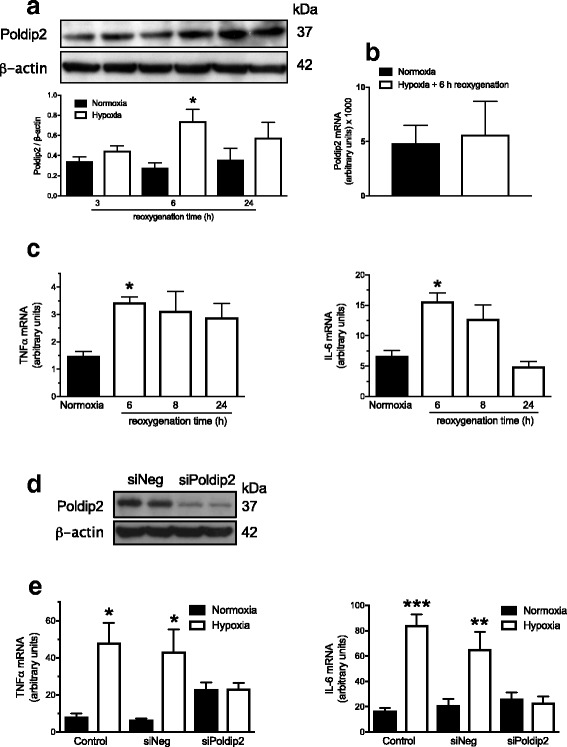
Poldip2 mediates cytokine upregulation by hypoxia in astrocytes. Astrocytes were cultured in control conditions or exposed to hypoxia and glucose deprivation for 4 h. Cells were returned to control conditions for the indicated times before extraction of protein or RNA. **a** Poldip2 protein expression was quantified by western blotting and densitometry. The bar graph represents means ± SEM of six independent experiments normalized to β-actin. Two-way ANOVA *p < 0.05 vs. normoxia. **b** Poldip2 mRNA was measured by quantitative RT-PCR. Bar graphs represent means ± SEM of ten independent experiments normalized to RPL. **c** TNFα and IL-6 mRNAs were measured by quantitative RT-PCR. Bar graphs represent means ± SEM of four independent experiments normalized to B2M. One-way ANOVA *p < 0.05 vs. normoxia. **d** Downregulation of Poldip2 protein after siRNA transfection was verified by western blotting. **e** Astrocytes were transfected with siNegative (siNeg, non-silencing control) or siPoldip2 before exposure to normoxic or hypoxic conditions (4 h), followed by recovery for 6 h. TNFα and IL-6 mRNAs were measured by quantitative RT-PCR. Bars represent means ± SEM of four independent experiments normalized to B2M. Two-way ANOVA *p < 0.05; **p < 0.01; ***p < 0.001 vs. normoxia

Activated astrocytes are important sources of inflammatory cytokines. We therefore used a model of cultured astrocytes exposed to OGD to mimic in vivo ischemia and test the possibility that Poldip2 might regulate cytokine induction in this cell type. Initially, we monitored Poldip2 protein expression following 4 h hypoxia and 3, 6, and 24 h reoxygenation. Similar to our in vivo findings, Poldip2 protein expression increased in response to hypoxia and 6 h reoxygenation (Fig. 4a). Poldip2 mRNA levels did not change under these conditions, suggesting that hypoxia/reoxygenation stimulates Poldip2 protein synthesis or inhibits its degradation (Fig. 4b).

To test whether or not the increase in Poldip2 protein expression correlates with increased TNF-α and IL-6 in response to hypoxia, the mRNA levels of those cytokines were monitored following a 4 h exposure to OGD followed by 6, 8, and 24 h reoxygenation. Figure 4c shows that, as expected, TNF-α and IL-6 mRNA levels were upregulated at 6 h reoxygenation. To delve into the connection between Poldip2 and astrocyte activation, and to determine whether Poldip2 can affect the astrocyte secretory phenotype, cells were transfected with siPoldip2 (small interfering RNA against Poldip2) or siNegative and subjected to 4 h OGD followed by 6 h reoxygenation before assessing IL-6 and TNF-α mRNA levels. Gene silencing was confirmed using immunoblotting (Fig. 4d). As is evident in Fig. 4e, downregulation of Poldip2 abrogated hypoxia-induced IL-6 and TNF-α upregulation in astrocytes.

### Poldip2 mediates IκB signaling pathway in vivo and in vitro

**Fig. 5:**
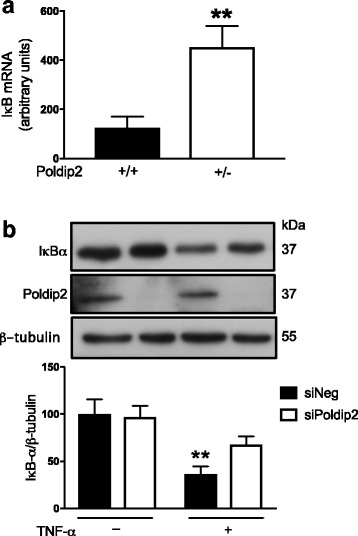
Poldip2 mediates IκB upregulation in vivo and in vitro. **a** Baseline IκB mRNA was evaluated by quantitative RT-PCR in uninjured brains from Poldip2^+/+^ and Poldip2^+/-^ mice. Bar graph represents means ± SEM from five mice per group normalized to GAPDH. **p < 0.01. **b** Astrocytes were transfected with siNegative (siNeg, non-silencing control) or siPoldip2 before exposure to TNF-α for 15 min. IκBα protein level was measured by western blotting and densitometry. Downregulation of Poldip2 after siRNA transfection was verified by western blotting. Bars represent means ± SEM of four to six independent experiments normalized to β-tubulin. Two-way ANOVA **p < 0.01 vs. siNeg no TNF-α

One of the most intriguing questions that arise from these observations involves the mechanism by which Poldip2 might exert its effects. Because NFκB is known to regulate transcription of both IL-6 and TNF-α [34], we hypothesized the loss of Poldip2 might inhibit the NFκB pathway. Consistent with this idea, we found increased mRNA levels of the NFκB inhibitor IκB in the brain tissue of Poldip2^+/−^ animals when compared to Poldip2^+/+^ mice (Fig. 5a). To determine whether Poldip2 regulates IκB degradation, we transfected cultured astrocytes cells with siNegative or siPoldip2 and evaluated IκBα protein degradation stimulated by TNF-α treatment. In astrocytes transfected with control siNegative, TNF-α treatment alone caused a significant decrease in IκBα when compared to control astrocytes. Of interest, siRNA against Poldip2 inhibited TNF-α-induced IκBα downregulation (Fig. 5b).

### Heterozygous deletion of Poldip2 abrogates cytokine induction in vivo

**Fig. 6:**
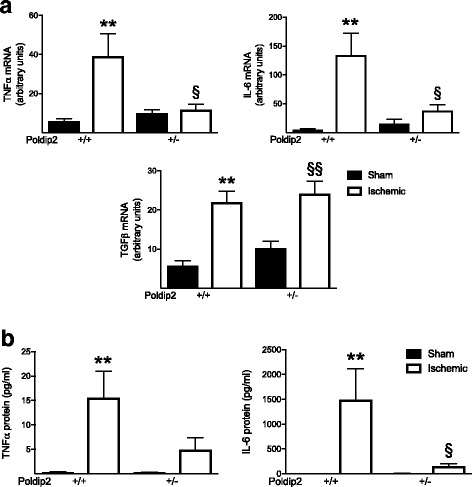
Poldip2 mediates cytokine upregulation induced by tMCAO. Cytokine mRNAs and proteins were measured in the brain hemispheres, 24 h after sham surgery or tMCAO in Poldip2^+/+^ and ^+/−^ mice. **a** TNF-α, IL-6, and TGF-β mRNAs were measured by quantitative RT-PCR. Bar graphs represent means ± SEM from six mice per group normalized to GAPDH. Two-way ANOVA **p < 0.01 vs. Poldip2^+/+^ sham mice, §*p* < 0.05 vs. Poldip2^+/+^ ischemic mice, and §§*p* < 0.01 vs. Poldip2^+/−^ sham mice. **b** TNF-α and IL-6 proteins were measured by multiplex high-sensitivity ELISA. Bar graphs represent means ± SEM from six mice per group. Two-way ANOVA **p < 0.01 vs. Poldip2^+/+^ sham mice and §*p* < 0.05 vs. Poldip2^+/+^ ischemic mice

Glial cells, including astrocytes, have been shown to release inflammatory cytokines in response to cerebral ischemia [35]. To confirm our observations in cultured astrocytes (Fig. 4e), we measured Poldip2-mediated cytokine release in vivo*.* Ischemic brain tissue was harvested 24 h after sham surgery or tMCAO and IL-6, TNF-α, and TGF-β mRNA levels were measured. As shown in Fig. 6, IL-6 and TNF-α mRNA levels were dramatically increased 24 h after reperfusion in the brains of Poldip2^+/+^ mice, but not in Poldip2^+/−^ animals (Fig. 6a). Similar results were found for MCP-1 and VEGF mRNA (Additional file 5: Figure S4). In contrast, ischemia-induced TGF-β mRNA expression was not affected by heterozygous deletion of Poldip2 (Fig. 6a). Corroborating the mRNA expression data, IL-6 and TNF-α protein levels were increased in the ischemic brain of Poldip2^+/+^ mice after tMCAO, but not in Poldip2^+/−^ mice (Fig. 6b).

### Poldip2 regulates MMP and TIMP mRNA expression in the ischemic brain following tMCAO

**Fig. 7:**
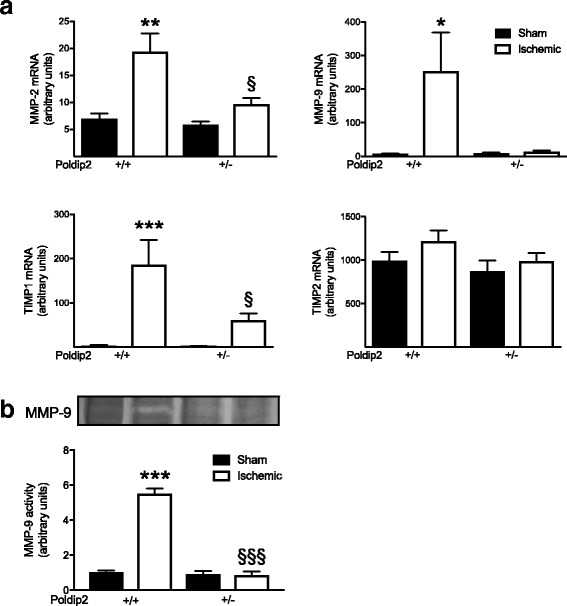
Poldip2 mediates MMP and TIMP1 upregulation in ischemic brain. **a** MMP-2, MMP-9, TIMP1, and TIMP2 mRNAs were measured by quantitative RT-PCR after sham surgery or tMCAO and 24 h reperfusion in ischemic and sham brain hemispheres of Poldip2^+/+^ and ^+/−^ mice. Bar graphs represent means ± SEM from six mice per group normalized to GAPDH. Two-way ANOVA *p < 0.05, **p < 0.01, and ***p < 0.001 vs. Poldip2^+/+^ sham mice and §*p* < 0.05 vs. Poldip2^+/+^ ischemic mice. **b** Representative gelatin zymography images and quantification of MMP-9 activity from ischemic and sham brain hemispheres in Poldip2^+/+^ and Poldip2^+/−^ mice. Bar graphs represent means ± SEM (*n* = 5 mice per group). Two-way ANOVA ***p < 0.001 vs. sham Poldip2^+/+^ mice §§§*p* < 0.001 vs. Poldip2^+/+^ ischemic mice

Cytokines upregulate MMPs, which then digest extracellular matrix proteins and essential components of tight junction proteins, thus compromising the BBB [11, 36]. To determine whether Poldip2 regulates MMP-2 and MMP-9 and their corresponding regulator tissue inhibitor of metalloproteinase TIMP2 and TIMP1, we examined their mRNA levels in Poldip2^+/+^ and Poldip2^+/−^ brain tissue after sham surgery or tMCAO. Both MMP-2 and MMP-9 mRNA were significantly elevated by tMCAO at 24 h in Poldip2^+/+^ vs. sham-operated mice, but not in Poldip2^+/−^ mice. Induction of TIMP1 mRNA by ischemia was also inhibited in Poldip2^+/−^ mice, but TIMP2 mRNA levels were largely unchanged (Fig. 7a). We further analyzed MMP activity using gelatin zymography. As shown in Fig. 7b, MMP-9 activity was increased in the brain tissue of Poldip2^+/+^ but not in Poldip2^+/−^ mice after tMCAO; MMP-2 activity levels were undetectable (data not shown).

### TNF-α partially reverses the effect of Poldip2 depletion

**Fig. 8:**
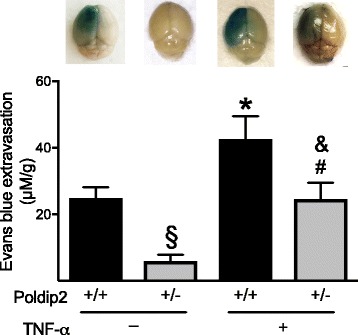
TNFα partially reverses the effect of Poldip2 depletion. Poldip2^+/+^ and Poldip2^+/−^ mice subjected to tMCAO received an intraperitoneal injection with or without 5 μg TNFα, immediately after tMCAO, followed by a 24 h reperfusion. Evans blue extravasation was measured as in Fig. 2. Bars represent means ± SEM from five to seven mice per group. Two-way ANOVA *p < 0.05 vs. Poldip2^+/+^ no TNFα; §*p* < 0.05 vs. Poldip2^+/+^ no TNFα; #*p* < 0.05 vs. Poldip2^+/+^ with TNFα and & *p* < 0.05 vs. Poldip2^+/−^ no TNFα

The observed inhibition of MMP expression and increase in BBB permeability in Poldip2^+/−^ mice could be a result of impaired induction of TNF-α or other cytokines. To begin to test this hypothesis, we administered TNF-α immediately after tMCAO to abrogate the loss of TNF-α in Poldip2^+/−^ mice, and permeability of the BBB was evaluated 24 h following tMCAO. As expected, Poldip2^+/+^ mice treated with TNF-α presented significantly increased permeability of the BBB when compared to Poldip2^+/+^ mice without TNF-α treatment (Fig. 8). TNF-α only partially reversed the effect of Poldip2 depletion on BBB permeability, suggesting that loss of Poldip2 affects downstream signaling pathways in addition to inhibiting TNF-α expression.

## Discussion

**Fig. 9:**
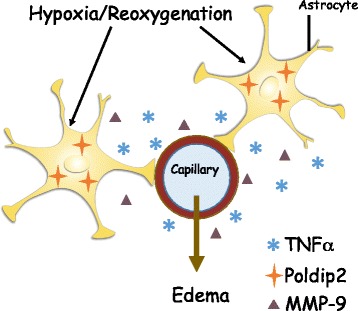
Poldip2 mediates hypoxia and reoxygenation-induced astrocyte activation following tMCAO. tMCAO induces hypoxia, thereby upregulating Poldip2 in astrocytes, leading to increased production of cytokines and a late phase breakdown of the blood-brain barrier

In this study, we report that Poldip2 has a profound and previously undiscovered role in cytokine release from the ischemic brain and regulation of BBB permeability. Poldip2 protein expression is upregulated in ischemic brain tissue following tMCAO, and astrocytes account for at least some of this increase, although Poldip2 is expressed in most other cell types in the brain as well. Poldip2 depletion nearly abolishes ischemia-induced late BBB permeability, possibly by regulating cytokine release and MMP activation. Our in vitro studies suggest that Poldip2 may exert these effects in astrocytes, a cell type that forms an integral part of the BBB. Importantly, this reduction in permeability is accompanied by an increase in survival and motor function (Fig. 2c, d). While infarct size is unchanged, this improvement in function suggests that either TTC staining is not sensitive enough to detect small changes in infarct volume or prevention of edema *per se* improves outcomes. These findings are consistent with the known roles of Poldip2 in vascular structure [7], hind limb ischemia [8], renal fibrosis [37], and protection against aortic aneurysms [7]. Our data thus suggest that Poldip2 represents a novel mediator of BBB function and dynamics at a late phase following cerebral ischemia, at least in part via cytokine release in astrocytes (Fig. 9).

We have previously demonstrated that Poldip2 is necessary for integrity and function of conduit arteries. Heterozygous deletion of Poldip2 was found to increase aortic extracellular matrix deposition and vascular stiffness and to impair phenylephrine and potassium chloride-induced contractility in isolated aortas [7]. In order to determine whether the improved response to cerebral ischemia was related to baseline structural and functional differences in Poldip2^+/−^ mice, micro-CT and electron microscopy studies were used to visualize the brain vasculature. A real-time investigation of cerebral blood flow dynamics was also performed. At the ultrastructural level, our analysis revealed no significant differences between the Poldip2^+/−^ and Poldip2^+/+^ mice. The quantitative analysis from both micro-CT and LDPI studies also revealed no differences between Poldip2^+/−^ mice and Poldip2^+/+^ littermates at the baseline level, except for a discrete increase in connectivity, which provides a foundation for future investigations.

Increased intracranial pressure with midline structure deviation is one of the predominant clinical implications of vasogenic edema, and it has been associated with higher mortality, longer hospitalization, and greater disability among stroke survivors [31]. Therefore, modulation of BBB dynamics following stroke is of interest as a druggable target to improve outcome. The BBB normally limits and regulates molecular exchange between the blood and the CNS [9]. Perivascular astrocytes respond to OGD by producing and releasing many active factors such as vascular endothelial growth factor (VEGF), MMPs, and pro-inflammatory cytokines to increase the vascular permeability [38–40]. TNF-α and IL-6 released by astrocytes are among the main pro-inflammatory cytokines implicated in the BBB dysfunction induced by stroke. A remarkable upregulation of TNF-α has been described to occur in the brain tissue following a stroke in both animal models and in patients [41, 42] which has been shown to induce cytoskeletal reorganization and decrease tight junction protein expression in endothelial cells [43, 44]. Increased plasma and cerebrospinal fluid levels of IL-6 seem to be correlated with stroke severity and poor clinical outcome in stroke patients [45, 46]. Importantly, both TNF-α and IL-6 are strongly implicated in the vascular permeability in rodents subjected to stroke [12, 47].

Our data demonstrate that Poldip2 protein expression is increased in reactive astrocytes in the ischemic brain as well as in astrocytes exposed to OGD and that loss of Poldip2 attenuates the upregulation of IL-6 and TNF-α both in vitro and in vivo. This likely occurs due to impaired degradation of IκB, the inhibitor of the major pro-inflammatory transcription factor NFκB (Fig. 5). To our knowledge, this is the first report linking Poldip2 to pro-inflammatory cytokines. Because the protection against BBB permeability in Poldip2^+/−^ mice is partially reversed by the restitution of TNF-α (Fig. 8), our data strongly suggest that one possible mechanism by which loss of Poldip2 prevents BBB permeability is via regulation of TNF-α secretion, although IL-6, MCP-1, and VEGF might also play a role. The fact that Poldip2 downregulation failed to abrogate induction of transforming growth factor-β suggests that Poldip2 specifically affects secretion of only certain cytokines following cerebral ischemia. Since the loss of Poldip2 remained partially protective even after repletion of TNF-α, it is likely that there are additional mechanisms involved in Poldip2-mediated BBB dynamics, such as direct effects on the integrity of tight junctions.

One important function of cytokine elevation is activation of MMPs. In acute stroke, MMPs degrade tight junction proteins and digest collagen type IV, facilitating blood cell extravasation and vasogenic edema [48]. Constitutively expressed MMPs, including MMP-2, seem to initiate the damage cascade early in the acute ischemic phase, and inducible MMPs, such as MMP-9, perpetuate the white matter and BBB damage over hours and days [49]. In the present study, we found an increase in both MMP-2 and MMP-9 mRNA and a clear increase in MMP-9 activity in Poldip-2^+/+^ mice but not in Poldip2^+/−^ mice, implying that reduction in MMP activity may contribute to the reduced BBB permeability in Poldip2^+/−^ mice after cerebral ischemia [50]. We made a similar observation in a hind limb ischemia model, where MMP-2 and MMP-9 activity were decreased in Poldip2^+/−^ mice when compared to Poldip2^+/+^ mice 21 days after ischemia [8]. Whether this reduction in MMP expression is a consequence of reduced cytokine release or is a direct effect of Poldip2 on MMP transcription remains to be determined.

After activation, MMPs are regulated mainly by TIMPs that can bind to the active site and block substrate availability. TIMP1 appears to be involved in inhibition of MMP-9, while TIMP2 seems to inhibit MMP-2 [49]. Wang et al. [51] reported an increase in TIMP1 mRNA in the ischemic cortex of rats after permanent MCAO, a finding in concordance with our observation of an elevation of TIMP1 24 h after tMCAO in Poldip2^+/+^ mice. This increase in TIMP1 may serve to inhibit or attenuate MMP-9 action in wild-type mice but is unnecessary in Poldip2^+/−^ mice where MMP-9 levels are not increased (Fig. 7). In contrast, we found no detectable differences in TIMP2 mRNA expression in either genotype 24 h following ischemia. Previous work suggests that TIMP2 activity was maximally increased only at 5 days after tMCAO [13], indicating that TIMP2 might have a role at a later time after the stroke onset. Importantly, the imbalance between TIMP2 and MMP-2 may determine the extent of extracellular matrix degradation and increased vascular permeability in Poldip2^+/+^ mice, a response that is absent in Poldip2^+/−^ mice.

The exact molecular mechanism underlying Poldip2-mediated effects on cytokines and MMPs remains to be fully elucidated. We previously showed that Poldip2, by virtue of its ability to bind p22phox, activates Nox4 to produce H_2_O_2_. Others have shown that deletion of Nox4 prevents BBB leakage [52], while pericyte-specific overexpression of Nox4 increases BBB permeability and infarct volume via activation of NFκB [53]. However, deletion of Nox4 was also neuroprotective while heterozygous deletion of Poldip2 selectively affected the BBB without affecting lesion volume. Thus, while Poldip2-mediated, Nox4-derived H_2_O_2_ may in part explain the role of Poldip2 in BBB integrity, other functions of Poldip2 likely have a role as well. The ability of Poldip2 depletion to upregulate IκB represents another candidate mechanism (Fig. 5). In addition, Poldip2 has a number of binding partners [54], verified and unverified, that may explain why loss of Poldip2 affects BBB permeability without affording neuroprotection. Nonetheless, protection against BBB permeability is likely to be physiologically important, given the decreased mortality and improved motor function in Poldip2^+/−^ mice compared to Poldip2^+/+^ mice 24 h after cerebral ischemia.

This study has some limitations. First, in our cell culture experiments, we only evaluated the role of Poldip2 in astrocytes and it is likely that Poldip2 in other cell types also contributes to the phenotype since it seems to be ubiquitously expressed in multiple cell types in the central nervous system. This possibility can be addressed when floxed Poldip2 mice are available. Second, we measured permeability changes only at 24 h, since measurement of Evans blue dye extravasation at earlier times can be variable. Given the multiple roles of Poldip2 in the vasculature, and the fact that it does not regulate lesion volume, we posit that Poldip2 also mediates early changes in permeability, but this will need to be proven experimentally. Third, replenishing TNF-α provides only suggestive evidence that this cytokine is involved in the mechanism by which Poldip2 affects the BBB, and other cytokines should be tested as well. Further studies will be necessary to identify the exact target of Poldip2 in the brain. Fourth, in vitro experiments were performed on an astrocyte cell line, rather than primary cells, although the concordance between in vitro and in vivo data makes this unlikely to be an issue. Finally, the therapeutic potential of Poldip2 as a target for modulating BBB permeability is complicated by the fact that homozygous Poldip2 deletion is embryonically lethal and significantly reduces the cell growth in isolated mouse embryonic fibroblasts [6]. However, because Poldip2 has many binding partners, it should be possible to design peptides or small molecules that specifically disrupt particular functions of Poldip2 while leaving others intact, thus modulating, but not eliminating Poldip2 function.

## Conclusions

In conclusion, we demonstrate that Poldip2 has a crucial role in increasing late BBB permeability following cerebral ischemia. Poldip2 depletion leads to decreased TNF-α and IL-6 secretion in the brain tissue and downregulation of MMPs and replenishing TNF-α partially reverses the impaired BBB permeability caused by Poldip2 depletion. We propose that Poldip2 is an important regulator of the disruption of the BBB in cerebral ischemia and represents a potentially druggable target to improve edema and mortality induced by stroke. Further studies of this novel protein may uncover new avenues for therapy of this debilitating condition.

## Additional files

Additional file 1: Figure S1. Characterization of the cerebral vasculature and blood flow. **a** Immediately after euthanasia, Poldip2^+/+^ and Poldip2^+/−^ mice were sequentially perfused with papaverine, formalin, and microfil compound containing lead chromate. The whole brain micro-CT scans were performed at 16-μm resolution. The bar graph represents vascular connectivity as means ± SEM of nine to ten mice per group. *p < 0.05. **b** Representative whole brain micro-CT angiographs from Poldip2^+/+^ and Poldip2^+/−^ mice. Imaging software was used to render 3D models, presented here as 2D maximal intensity projections. **c** Baseline cerebral blood flow (CBF) flux measured using LDPI. The bar graph represents means ± SEM of three to four mice per group. **d** Anesthetized Poldip2^+/+^ and Poldip2^+/−^ mice were endotracheally intubated, and hypercapnia was induced three successive times using 5% CO_2_ inhalation for 5 min, separated with 5 min normocapnia intervals. Cerebrovascular reactivity (CVR) was calculated as the increase of CBF (%) divided by the maximum increase in end-tidal CO_2_ pressure (∆mmHg) during hypercapnia. The bar graph represents means ± SEM of three to four mice per group. **e** Representative LDPI tracings from Poldip2^+/+^ and Poldip2^+/−^ mice. **f** Representative electron micrographs from cortical capillaries of Poldip2^+/+^ and Poldip2^+/−^ mice. Lumen (L); endothelial cells (EC); basement membrane (BM); astrocyte (As), and pericyte (P). Scale bar 1 μm. (PDF 1588 kb)

Additional file 2: Figure S2. Poldip2 co-localizes with cortical perivascular astrocytes. Poldip2 staining in cortical perivascular astrocytes. Tissue sections including blood vessels were prepared from uninjured Poldip2^+/+^ mice. Sections were stained for immunofluorescence with primary antibodies specific for Poldip2 (purple), endothelial cells (Isolectin IB_4_, green), or the astrocyte marker GFAP (red). Nuclei were stained with DAPI (blue). Images are representative of four independent experiments. Scale bar 7 μm. (PDF 5668 kb)

Additional file 3: Video S1. Co-localization of Poldip2 with cortical perivascular astrocytes. A tissue section was prepared from an uninjured Poldip2^+/+^ mouse and stained for immunofluorescence with primary antibodies specific for Poldip2 (purple), endothelial cells (Isolectin IB_4_, green), or the astrocyte marker GFAP (red). Nuclei were stained with DAPI (blue). Z-stack images were collected and animated in 3D using Imaris software to produce a video file. (MP4 3146 kb)

Additional file 4: Figure S3. Poldip2 deletion reduces blood-brain barrier disruption 24 h after non-reperfusion cerebral ischemia induced by temporary unilateral carotid ligation and hypoxia. Evans blue extravasation was measured as in Fig. 2. The bar graph represents means ± SEM of three to five mice per group. One-way ANOVA *p < 0.05. (PDF 96 kb)

Additional file 5: Figure S4. Poldip2 mediates MCP-1 and VEGF upregulation induced by tMCAO. Cytokine mRNAs were measured in ischemic and sham brain hemispheres, 24 h after sham surgery or tMCAO and 24 h reperfusion in Poldip2^+/+^ and Poldip2^+/−^ mice. MCP-1 (**a**) and VEGF (**b**) mRNAs were measured by quantitative RT-PCR. Bar graphs represent means ± SEM from five to six mice per group normalized to GAPDH. Two-way ANOVA *p < 0.05 vs. sham mice. (PDF 125 kb)

## Abbreviations

NFκB: Nuclear factor kappa B
B2M: Beta-2-microglobulin
BBB: Blood-brain barrier
CP: Cerebral perfusion
GFAP: Glial fibrillary acidic protein
IL-6: Interleukin 6
MCP-1: Monocyte chemotactic protein-1
MMP: Matrix metalloproteinase
Nox4: NADPH oxidase 4
OGD: Oxygen and glucose deprivation
Poldip2: Polymerase (DNA-directed) delta-interacting protein 2
RPL: Ribosomal protein L13a
siNeg: Negative control small interfering RNA
siRNA: Small interfering RNA
TGF-β: Transforming growth factor beta
TIMP: Tissue inhibitor of metalloproteinase
tMCAO: Transient middle cerebral artery occlusion
TNF-α: Tumor necrosis factor alpha
VEGF: Vascular endothelial growth factor
