# Supplementary figures and images for: Polymerase delta-interacting protein 2 deficiency protects against blood-brain barrier permeability in the ischemic brain
Source: J Neuroinflammation. 2018 Feb 17;15:45. doi: 10.1186/s12974-017-1032-1 (PMC5816395; doi:10.1186/s12974-017-1032-1)

Additional file 1: Figure S1

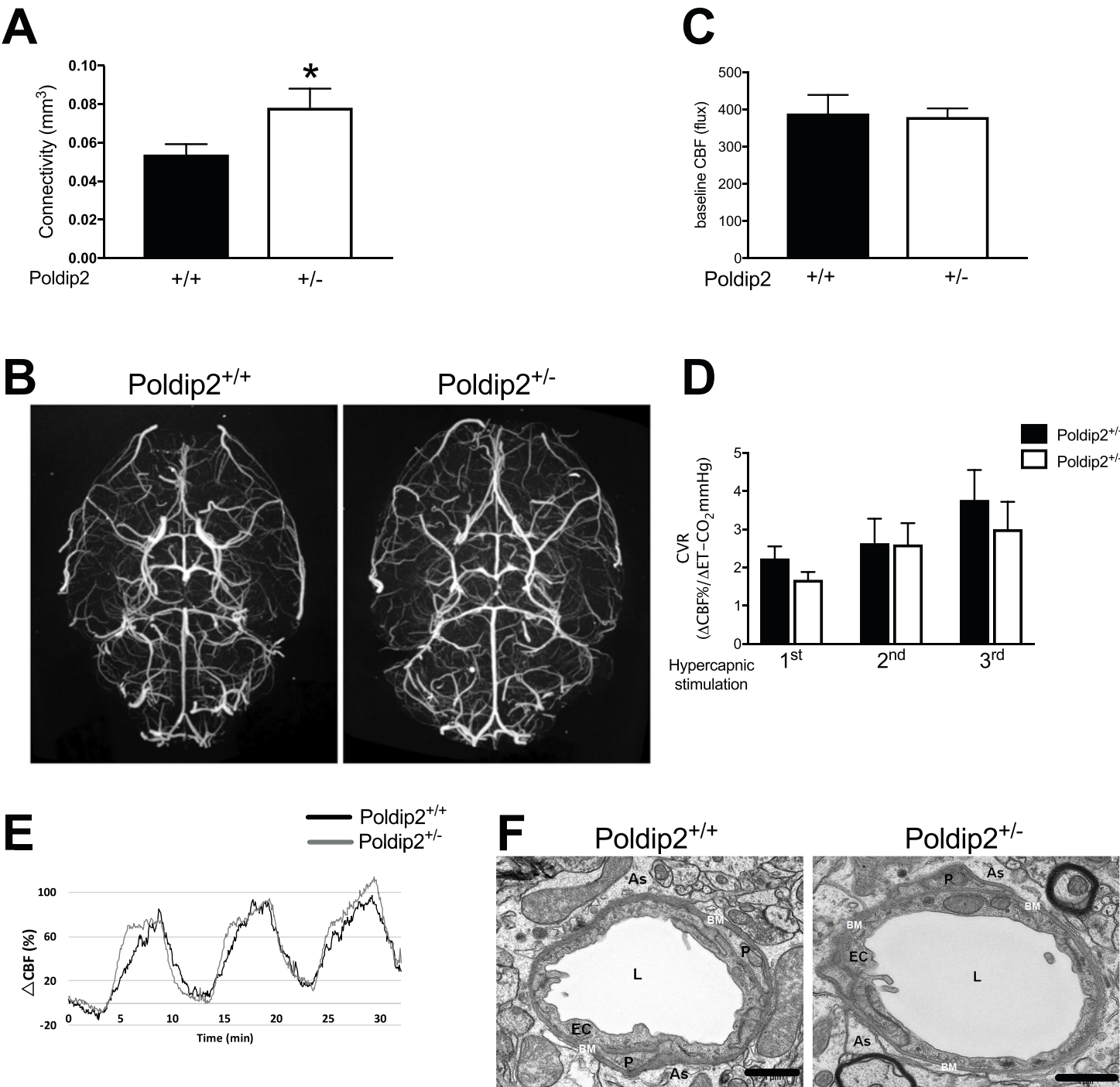

Supplement: Supplementary file 1 — Characterization of the cerebral vasculature and blood flow. a Immediately after euthanasia, Poldip2+/+ and Poldip2+/− mice were sequentially perfused with papaverine, formalin, and microfil compound containing lead chromate. The whole brain micro-CT scans were performed at 16-μm resolution. The bar graph represents vascular connectivity as means ± SEM of nine to ten mice per group. *p < 0.05. b Representative whole brain micro-CT angiographs from Poldip2+/+ and Poldip2+/− mice. Imaging software was used to render 3D models, presented here as 2D maximal intensity projections. c Baseline cerebral blood flow (CBF) flux measured using LDPI. The bar graph represents means ± SEM of three to four mice per group. d Anesthetized Poldip2+/+ and Poldip2+/− mice were endotracheally intubated, and hypercapnia was induced three successive times using 5% CO2 inhalation for 5 min, separated with 5 min normocapnia intervals. Cerebrovascular reactivity (CVR) was calculated as the increase of CBF (%) divided by the maximum increase in end-tidal CO2 pressure (∆mmHg) during hypercapnia. The bar graph represents means ± SEM of three to four mice per group. e Representative LDPI tracings from Poldip2+/+ and Poldip2+/− mice. f Representative electron micrographs from cortical capillaries of Poldip2+/+ and Poldip2+/− mice. Lumen (L); endothelial cells (EC); basement membrane (BM); astrocyte (As), and pericyte (P). Scale bar 1 μm. (PDF 1588 kb) [file 12974_2017_1032_MOESM1_ESM.pdf]

Additional file 2: Figure S2

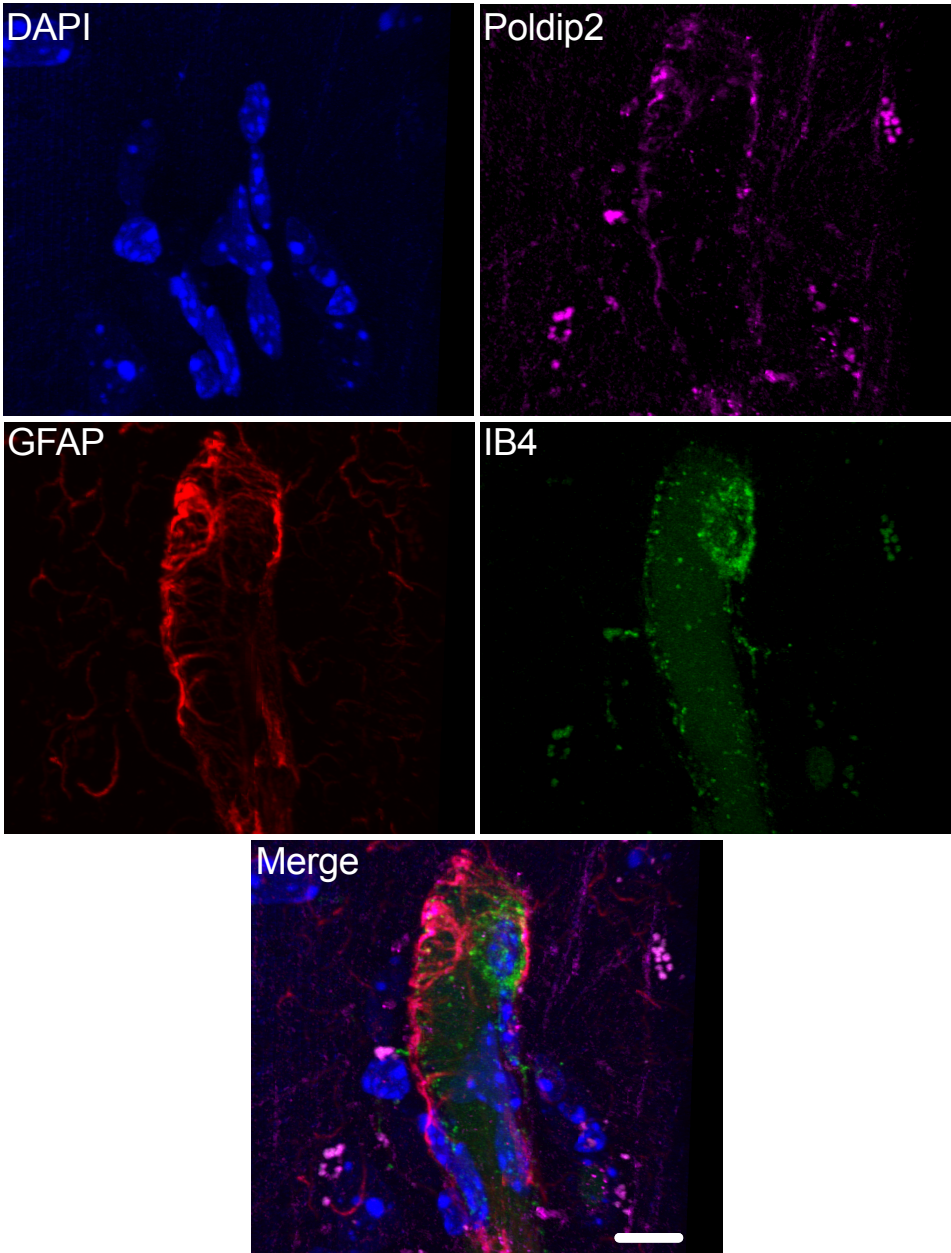

Supplement: Supplementary file 2 — Poldip2 co-localizes with cortical perivascular astrocytes. Poldip2 staining in cortical perivascular astrocytes. Tissue sections including blood vessels were prepared from uninjured Poldip2+/+ mice. Sections were stained for immunofluorescence with primary antibodies specific for Poldip2 (purple), endothelial cells (Isolectin IB4, green), or the astrocyte marker GFAP (red). Nuclei were stained with DAPI (blue). Images are representative of four independent experiments. Scale bar 7 μm. (PDF 5668 kb) [file 12974_2017_1032_MOESM2_ESM.pdf]

Additional file 4: Figure S3

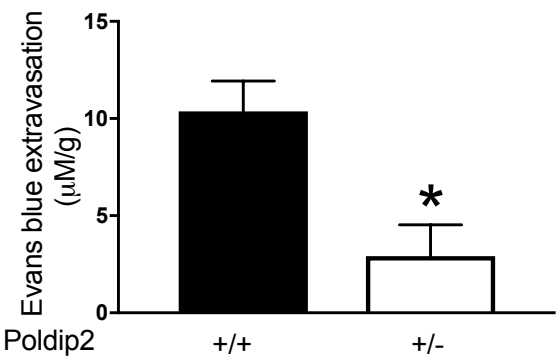

Supplement: Supplementary file 4 — Poldip2 deletion reduces blood-brain barrier disruption 24 h after non-reperfusion cerebral ischemia induced by temporary unilateral carotid ligation and hypoxia. Evans blue extravasation was measured as in Fig. 2. The bar graph represents means ± SEM of three to five mice per group. One-way ANOVA *p < 0.05. (PDF 96 kb) [file 12974_2017_1032_MOESM4_ESM.pdf]

## Additional file 5: Figure S4

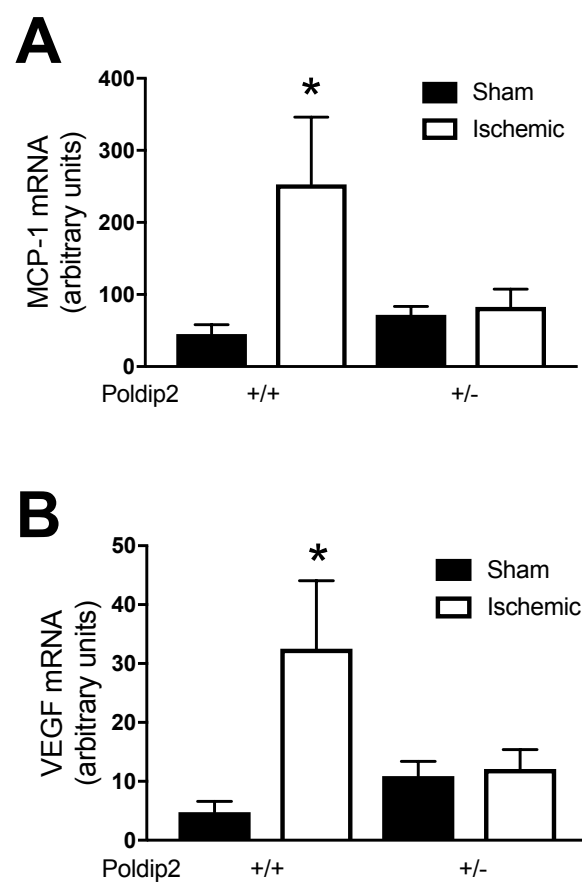

Supplement: Supplementary file 5 — Poldip2 mediates MCP-1 and VEGF upregulation induced by tMCAO. Cytokine mRNAs were measured in ischemic and sham brain hemispheres, 24 h after sham surgery or tMCAO and 24 h reperfusion in Poldip2+/+ and Poldip2+/− mice. MCP-1 (a) and VEGF (b) mRNAs were measured by quantitative RT-PCR. Bar graphs represent means ± SEM from five to six mice per group normalized to GAPDH. Two-way ANOVA *p < 0.05 vs. sham mice. (PDF 125 kb) [file 12974_2017_1032_MOESM5_ESM.pdf]
